# In‐depth proteomics approach reveals novel biomarkers of cardiac remodelling after myocardial infarction: An exploratory analysis

**DOI:** 10.1111/jcmm.15611

**Published:** 2020-07-23

**Authors:** Shuai Mao, Yubin Liang, Peipei Chen, Yuzhuo Zhang, Xin Yin, Minzhou Zhang

**Affiliations:** ^1^ Key Discipline of Integrated Chinese and Western Medicine Second Clinical College Guangzhou University of Chinese Medicine Guangzhou China; ^2^ Department of Critical Care Medicine Guangdong Provincial Hospital of Chinese Medicine Guangzhou China

**Keywords:** antibody array, cardiac remodelling, myocardial infarction, predictor

## Abstract

Cardiac remodelling following myocardial infarction (MI) is a maladaptive change associated with progressive heart failure and compromises long‐term clinical outcome. A substantial proportion of patients afflicted by MI still develop adverse outcomes associated with cardiac remodelling. Therefore, it is crucial to identify biomarkers for the early prediction of cardiac remodelling. An in‐depth proteomics approach, including both semi‐quantitative and quantitative antibody arrays, was used to identify circulating biomarkers that may be associated with detrimental cardiac remodelling. Furthermore, statistical correlation analysis was performed between the candidate biomarkers and clinical cardiac remodelling data to demonstrate their clinical utility. A systematic proteomics approach revealed that sclerostin (SOST), growth differentiation factor‐15 (GDF‐15), urokinase‐type plasminogen activator (uPA), and midkine (MK) were increased, while monocyte chemotactic protein‐3 (MCP‐3) was uniquely decreased in MI patients who developed cardiac remodelling, compared to MI patients who did not develop cardiac remodelling and healthy humen. Moreover, correlation analyses between serum proteomes and cardiac remodelling echocardiographic parameters demonstrated a moderate positive association between left ventricular end‐diastolic volume index (LVEDVi) and the three serum proteins, uPA, MK and GDF‐15 (*P* < .05, respectively), and a moderate negative correlation between LV ejection fraction (LVEF) and these serum proteins (*P* < .05, respectively). Importantly, uPA and MK were firstly identified to be associated with the development of cardiac remodelling. The present study contributes to a better understanding of the various cytokines expressed during adverse cardiac remodelling. The identified biomarkers may facilitate early identification of patients at high risk of ischaemic heart failure pending further confirmation through larger clinical trials.

## 1 INTRODUCTION

Cardiac remodelling after acute myocardial infarction (MI) is characterized by progressive expansion of infarcted myocardium and disproportionate alterations in the geometry and function of the ventricular chamber.^1^ The pathophysiological process of cardiac remodelling starts immediately after an acute MI and, if not attenuated or reversed through effective interventions, results in poor clinical prognosis, including ventricular arrhythmias, heart failure and subsequent cardiovascular mortality.^2^, ^3^ Moreover, once cardiac remodelling manifests with clinical heart failure symptoms, standard pharmacotherapies including angiotensin receptor‐neprilysin inhibitor, natriuretic peptides, angiotensin‐converting enzyme inhibitors, or β‐blockers have limited effect on reversing the remodelling and/or improving the clinical symptoms.^4^

Cardiovascular magnetic resonance imaging is the gold standard for diagnosing cardiac remodelling despite being cumbersome and complex, and leading to increased risk of adverse cardiovascular events during the examination.^5^ Hence, this technique is sparingly used in clinical setting. Currently, transthoracic echocardiography has been widely available to assess the segmental and overall cardiac structure and function. However, both cardiac imaging measurements do not evaluate the complex process of biochemical and neuro‐endocrine changes which are at the core of the vicious cycle termed “infarct remodelling”.^6^ So far, N‐terminal pro‐brain natriuretic peptide (NT‐proBNP) is established as useful non‐invasive indicator of cardiac remodelling and dysfunction.^7^, ^8^, ^9^ Elevated cardiac troponins are demonstrated as markers of increased risk for progressive left ventricular (LV) dysfunction in chronic heart failure.^10^, ^11^ Soluble suppression of tumorigenicity 2 is a predictor of reverse left ventricular remodelling.^12^, ^13^ Nevertheless, there are no early and effective means to predict the occurrence of remodelling following MI. Therefore, the development of novel and potentially more accurate approaches to predict cardiac remodelling after MI is urgently needed.

Novel biomarkers are crucial for understanding the pathophysiology, early diagnosis and prognosis of a disease, as well as for developing effective personalized pharmacological management therapies. There are several promising tools to identify such biomarkers, including clinical proteomics, an approach dedicated to the global study of secreted proteins from organs throughout the body, which may contain biomarkers for prognostication and treatment response.^14^ Currently, there are two main strategies for analysing plasma/serum proteome, including microarray assays and mass spectrometry. Regrettable, mass spectrometry suffers from high false‐positive rates and poor sensitivity.^15^ Samples need to be treated for the remove of high‐abundant proteins when detected by mass spectrometry, which would cause the depletion of low‐abundant proteins such as inflammatory, angiogenic cytokines, chemokines, growth factors and so on playing a very important role in the pathophysiology of diseases. Some studies showed that mass spectrometry is valuable at the detection of lipid proteins and enzymes, whereas antibody array could detect cytokines, chemokines and receptors.^16^, ^17^ In particular, the antibody microarrays are recognized as an innovative and preferential technology for the identification of circulating cytokine biomarkers, with the advantages of being high‐throughput amenable and highly sensitive for phenotype assessment.^18^

In the present study, the antibody microarray technology was used to identify the serum proteome of MI patients with cardiac remodelling development. Furthermore, statistical correlation analysis was preformed between candidate cytokines and clinical cardiac remodelling data to validate the biomarkers. The comprehensive analysis of the proteome may help to better understand the pathogenesis of cardiac remodelling as well as to identify more novel serum biomarkers for early and more accurate prediction of this pathological process.

## 2 MATERIALS AND METHODS

### 2.1 Study design and participants

**FIGURE 1:**
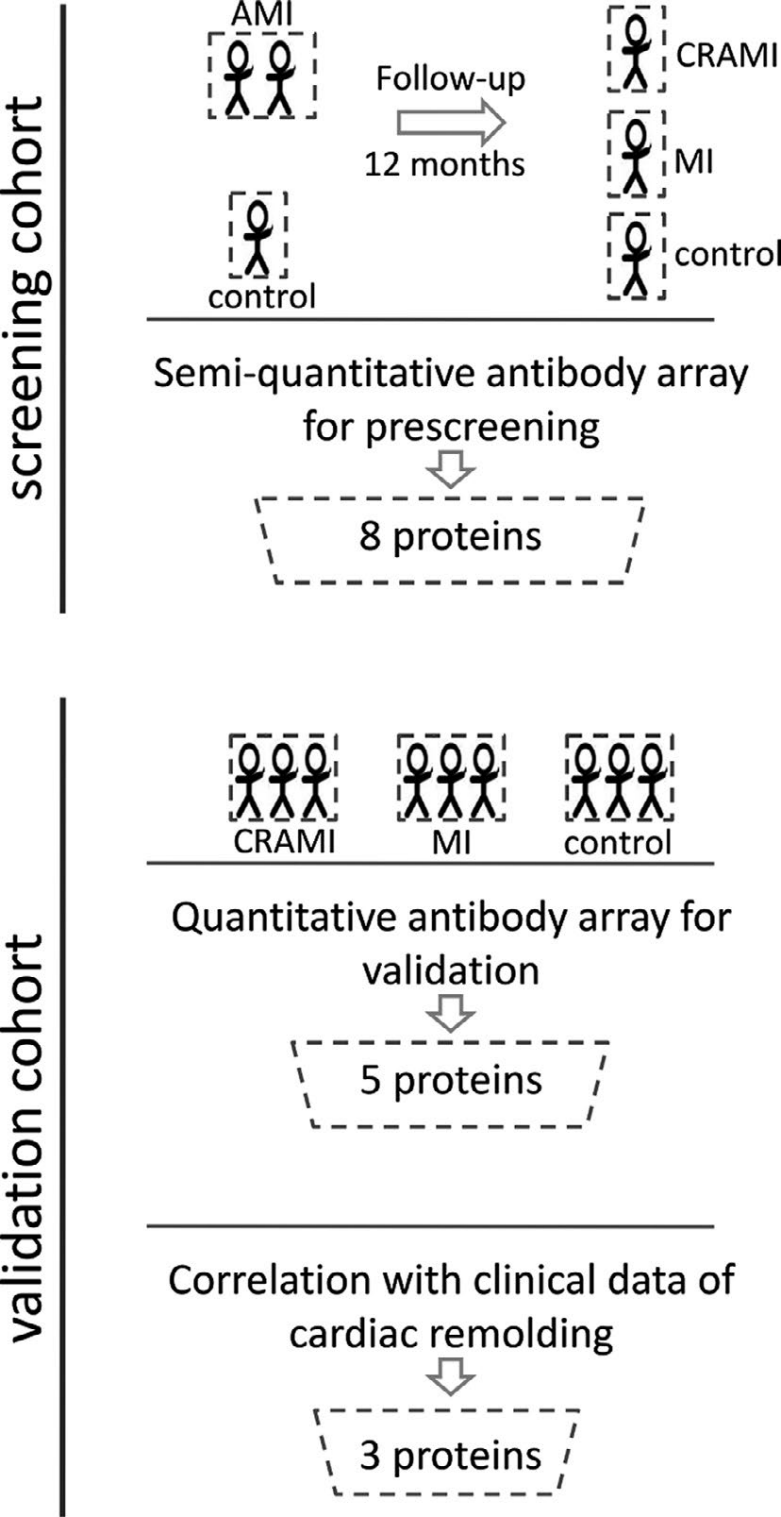
Schematic of the screening strategy. Identification of serum proteomic biomarkers for prediction of cardiac remodelling following myocardial infarction. CRAMI group, cardiac remodelling after myocardial infarction; MI group, myocardial infarction without cardiac remodelling

The study was conducted in accordance with the Declaration of Helsinki and its text revisions, and all participants provided informed consent. The Ethics Committee of Guangdong Provincial Hospital of Chinese Medicine approved the trial design (B2015‐129‐01). Figure 1 illustrates the experimental workflow of the study.

The study population consisted of patients diagnosed with acute ST‐elevation acute myocardial infarction (STEMI) according to the American College of Cardiology/American Heart Association guidelines.^19^ For the screening cohort, serum samples were obtained within the first 24 hours (baseline) after acute MI diagnosis. All the patients were treated with standard therapy recommended by the international guidelines.^19^ After a 12‐month follow‐up, some MI patients developed LV remodelling (defined as >20% change in LV end‐diastolic volume index [LVEDVi] assessed by echocardiography) and were assigned into cardiac remodelling after myocardial infarction (CRAMI) group.^20^ While the MI patients without cardiac remodelling development were assigned into MI group. Exclusion criteria included previous MI, documented malignant arrhythmia, cardiac shock, or depressed LV ejection fraction <15%, taking medications affecting cardiac remodelling, hepatic or renal impairment, active pregnancy, or a life expectancy of <1 year. A matched population with no evidence of cardiovascular disease, excluded by a comprehensive physical examination and an echocardiogram, served as the control group. There were no significant differences in age, sex, ethnicity and cardiac function among the three groups at baseline. The clinical parameters and medical history of each participant at 12‐month follow‐up are summarized in Table 1.

**TABLE 1:** Participant characteristics for pre‐screening

	CRAMI (*n* = 3)	MI (*n* = 3)	Control (*n* = 3)
Age (years)	62.85 ± 6.95	63.00 ± 8.06	62.23 ± 5.98
BMI (kg/m^2^)	25.52 ± 3.22	24.82 ± 3.01	25.11 ± 2.51
Heart rate (beats/min)	77.08 ± 11.38	76.16 ± 10.63	76.54 ± 11.25
Systolic blood pressure (mmHg)	128.18 ± 16.26	130.66 ± 16.70	131.31 ± 13.42
NT‐proBNP peak (ng/L)	1023.56 ± 231.91	234.31 ± 58.76	45.21 ± 13.63
LVEDVi, mL/m^2^	59.89 ± 6.06	52.47 ± 5.68	51.19 ± 5.71
LVESVi, mL/m^2^	32.97 ± 5.34	31.19 ± 5.71	31.33 ± 3.24
LVEF, %	42.71 ± 10.02	54.11 ± 11.15	55.73 ± 5.71

Abbreviations: CRAMI, cardiac remodelling after myocardial infarction; LVEDVi, left ventricular end‐diastolic volume index, LVESVi, left ventricular end‐systolic volume index; LVEF, left ventricular ejection fraction; MI, myocardial infarction without cardiac remodelling; NT‐proBNP, N‐terminal pro‐brain natriuretic peptide.

For validation cohort, the serum samples were obtained from MI patients with (*n* = 29) or without (*n* = 25) LV remodelling according to the aforementioned inclusion and exclusion criteria. Furthermore, healthy people matched with our observed patients served as the control group (*n* = 12). The clinical parameters of each group in validation cohort are summarized in Table 2.

**TABLE 2:** Participant characteristics for validation

	CRAMI (*n* = 29)	MI (*n* = 25)	Control (*n* = 12)
Age (years)	63.12 ± 9.01	63.23 ± 10.12	64.11 ± 5.98
BMI (kg/m^2^)	25.01 ± 2.93	24.94 ± 3.23	24.32 ± 1.05
Heart rate (beats/min)	75.08 ± 11.09	75.32 ± 9.33	75.12 ± 11.25
Systolic blood pressure (mmHg)	125.22 ± 13.65	125.87 ± 12.75	126.19 ± 11.89
NT‐proBNP peak (ng/L)	1241.73 ± 271.22	254.49 ± 78.54	50.32 ± 11.28
LVEDVi, mL/m^2^	61.25 ± 6.86	52.81 ± 5.11	50.34 ± 7.29
LVESVi, mL/m^2^	33.01 ± 6.86	30.22 ± 5.54	30.43 ± 7.06
LVEF, %	34.71 ± 10.53	55.04 ± 10.12	54.56 ± 5.09

Abbreviations: CRAMI, cardiac remodelling after myocardial infarction; LVEDVi, left ventricular end‐diastolic volume index, LVESVi, left ventricular end‐systolic volume index; LVEF, left ventricular ejection fraction; MI, myocardial infarction without cardiac remodelling; NT‐proBNP, N‐terminal pro‐brain natriuretic peptide.

### 2.2 Echocardiographic measurements

All echocardiographic measurements were performed by an independent cardiac sonographer who was blinded to the allocation. A commercially available ultrasound system (Philips Medical Systems) was used to obtain and store the images for subsequent off‐line analysis. Standard two‐dimensional short‐axis view of the LV was obtained at the level of the papillary muscle to record M‐mode tracings. LV end‐diastolic/end‐systolic internal diameter and LV anterior wall thickness were measured. Biplane end‐diastolic and systolic volumes are calculated according to the modified Simpson's rule. All measurements were obtained and averaged over three consecutive cardiac cycles.

### 2.3 Semi‐quantitative antibody array for pre‐screening

A Human Cytokine Antibody Array (GSH‐CAA‐440, RayBiotech Company) that simultaneously and semi‐quantitatively detects 440 cytokines in a single experiment was used according to the manufacturer's instructions.^3^ Briefly, after a dilution with blocking buffer (1:1), serum samples (Table 1) were incubated with 440 capture antibodies previously coated onto glass slides overnight. After washing, a biotin‐conjugated anti‐cytokine antibody mix was added into the arrays and further incubated for 2 hours. Finally, Cy3‐conjugated streptavidin was used to detect the intensities of the arrays which were subsequently exposed using an InnoScan 300 Microarray Scanner (Innopsys). The signal values of the 440 cytokines were normalized using a factor (the positive control values from certain array/the positive control values from the array whose 440 cytokine signal values are normalized) via RayBiotech GSH‐CAA‐440 analysis tool by Microsoft Excel technology.

### 2.4 Quantitative antibody array for validation

To validate the differential cytokines screened from the high‐throughput array GSH‐CAA‐440, a custom Human Cytokine Antibody Array (QAH‐CUST‐39, RayBiotech Company) that can simultaneously and quantitatively detect thirty‐nine cytokines was used to detect more serum samples (Table 2). Briefly, the standards (gradient dilution) and serum samples (diluted two times) were added into the array pools for overnight incubation. Then, a biotin‐conjugated anti‐cytokine antibody mix and Cy3‐conjugated streptavidin were successively added into the array pools followed by 2 hours of incubation. The slides were scanned using an InnoScan 300 Microarray Scanner (Innopsys). Finally, the concentration of the thirty‐nine cytokines in the serum was calculated using their signal values and the standard curve.

### 2.5 Statistical analysis

Comparisons between groups were performed by one‐way ANOVA followed by multiple comparisons performed with post hoc Bonferroni test using SPSS v20 (IBM Corp.). The relationship between serum proteome and cardiac remodelling echocardiographic data was calculated using bivariate correlation analysis. Differences were considered statistically significant when *P* values were < .05. All data are shown as mean ± SD (standard deviation). In addition, fold change (FC) between groups was calculated to indicate the relative levels of the cytokines.

## 3 RESULTS

### 3.1 Differential proteins analysis

To identify the specific proteins involved in cardiac remodelling following MI, the antibody array data of the three groups were analysed by one‐way ANOVA. The Bonferroni post hoc analysis showed that forty‐one cytokines were differentially expressed between the CRAMI and control group, eighteen cytokines were differentially expressed between the CRAMI and MI group, and seventeen cytokines were differentially expressed between the MI and control groups with *P* values < .05 (Table S1).

### 3.2 Analysis of specific biomarkers of cardiac remodelling after MI

**FIGURE 2:**
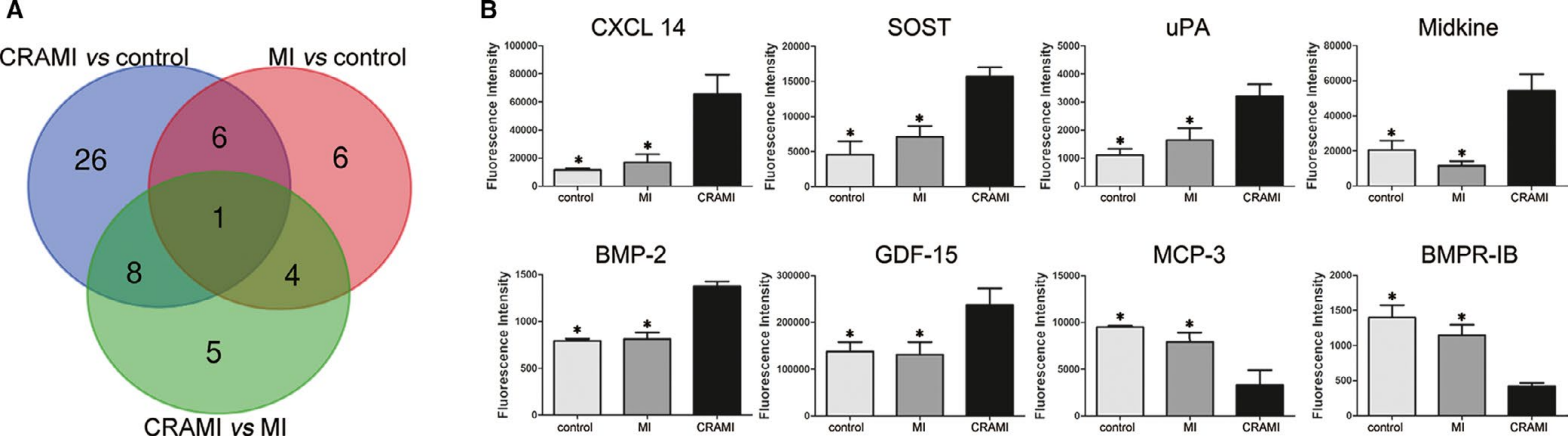
Proteins screened by semi‐quantitative antibody array. A, Venn diagram analysis of proteins differentially expressed between any two groups from the semi‐quantitative antibody array pre‐screen identified eight proteins simultaneously differential between CRAMI and MI group and between CRAMI and control group, but not differential between MI and control group, which are defined as specific biomarkers of cardiac remodelling after MI. B, The levels of these eight specific proteins of cardiac remodelling after MI are shown by histogram analysis using their fluorescence signal values from the semi‐quantitative antibody array from the three groups. * means *P* < .05 when compared to CRAMI group. CRAMI group, cardiac remodelling after myocardial infarction; MI group, myocardial infarction without cardiac remodelling

**FIGURE 3:**
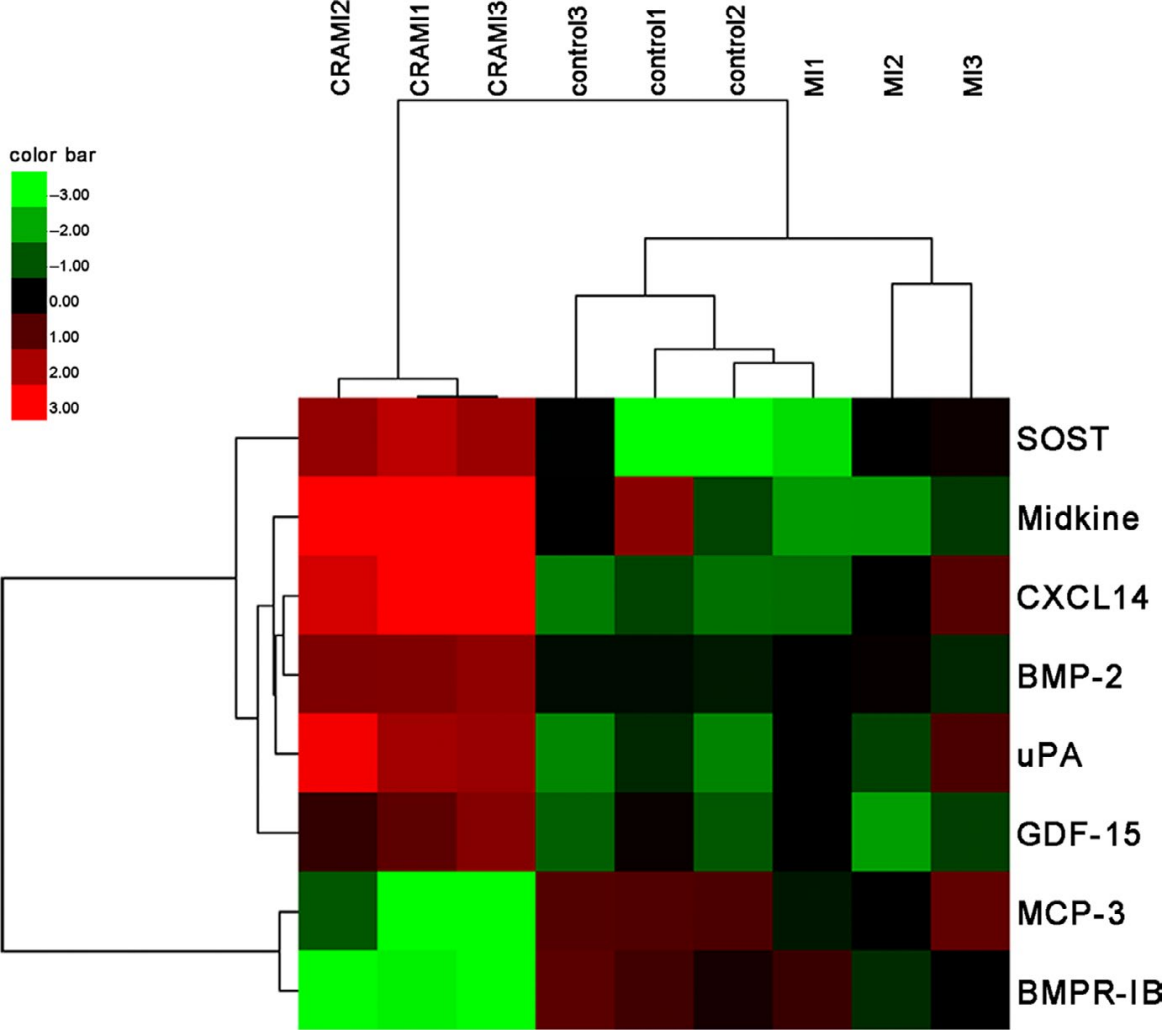
Unsupervised‐hierarchical clustering analysis. The fluorescence signal values of these eight specific proteins of cardiac remodelling after MI from semi‐quantitative antibody array were used for unsupervised‐hierarchical cluster analysis. Green indicates low levels of the proteins, black for median levels, and red for high levels. CRAMI group, cardiac remodelling after myocardial infarction; MI group, myocardial infarction without cardiac remodelling

As per definition, the specific biomarkers of cardiac remodelling following MI would be the proteins that show differential expression between CRAMI and MI group as well as between CRAMI and control group, but not between MI and control group. Therefore, a Venn diagram analysis was performed and eight specific biomarkers of cardiac remodelling following MI were identified (Figure 2A). As shown in Table 3, the specific biomarkers included bone morphogenetic protein‐2 (BMP‐2), sclerostin (SOST), chemokine ligand 14 (CXCL14), growth differentiation factor‐15 (GDF‐15), urokinase‐type plasminogen activator (uPA), monocyte chemotactic protein‐3 (MCP‐3), midkine (MK) and bone morphogenetic protein receptor IB (BMPR‐IB). Furthermore, a boxplot analysis showed that BMP‐2, SOST, CXCL14, GDF‐15, uPA and MK were increased in CRAMI group as compared to MI and control groups, while MCP‐3 and BMPR‐IB were decreased (Figure 2B). An unsupervised‐hierarchical clustering using the signal values of these eight proteins could distinguish CRAMI group from the other two groups with 100% accuracy (Figure 3).

**TABLE 3:** Specific biomarkers of cardiac remodelling after myocardial infarction from semi‐quantitative antibody array assay

	Control group	MI group	CRAMI group	MI vs Control		CRAMI vs Control		CRAMI vs MI	
	Mean ± SD	Mean ± SD	Mean ± SD	*P* value	FC	*P* value	FC	*P* value	FC
CXCL14	11 538.6 ± 1344.5	16 894.9 ± 5842.9	65 691.0 ± 25 125.7	1.000	1.464	.003	5.693	.006	3.888
SOST	4554.9 ± 3435.5	7070.8 ± 2899.0	15 710.6 ± 1282.5	.690	1.552	.006	3.449	.025	2.222
uPA	1099.9 ± 231.3	1636.7 ± 427.0	3200.1 ± 631.9	1.000	1.488	.035	2.909	.027	1.955
MK	20 484.7 ± 8254.4	11 570.1 ± 2525.4	54 368.9 ± 9238.0	.308	0.565	.003	2.654	.021	4.699
BMP‐2	793.2 ± 22.5	812.5 ± 69.7	1378.9 ± 48.3	1.000	1.024	.000	1.738	.000	1.697
GDF‐15	137 579.4 ± 31 065.9	131 192.1 ± 37 333.9	237 457.8 ± 35 148.8	1.000	0.954	.009	1.726	.017	1.810
MCP‐3	9491.9 ± 152.7	7896.2 ± 1956.6	3313.8 ± 1576.9	.905	0.832	.006	0.349	.019	0.420
BMPR‐IB	1399.3 ± 174.2	1147.0 ± 216.3	422.7 ± 42.2	.591	0.820	.004	0.302	.001	0.369

Abbreviations: CRAMI, cardiac remodelling after myocardial infarction; MI, myocardial infarction without cardiac remodelling.

### 3.3 Validation of specific biomarkers

**FIGURE 4:**
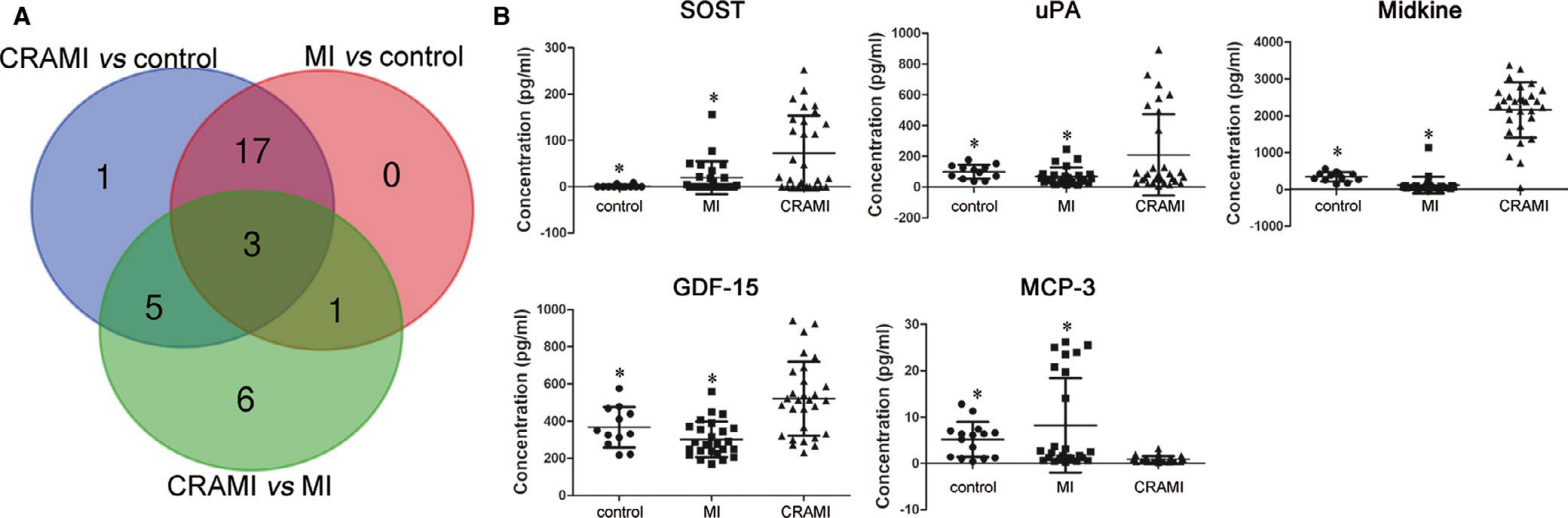
Proteins validated by quantitative antibody array. A, Results of the quantitative antibody array were validated using Venn diagram analysis, and five proteins simultaneously differential between CRAMI and MI group and between CRAMI and control group, but not differential between MI and control group were identified. B, The concentration of the five specific proteins of cardiac remodelling after MI validated by the quantitative antibody array is shown using a scatter diagram analysis. * means *P* < .05 when compared to CRAMI group. CRAMI group, cardiac remodelling after myocardial infarction; MI group, myocardial infarction without cardiac remodelling

**FIGURE 5:**
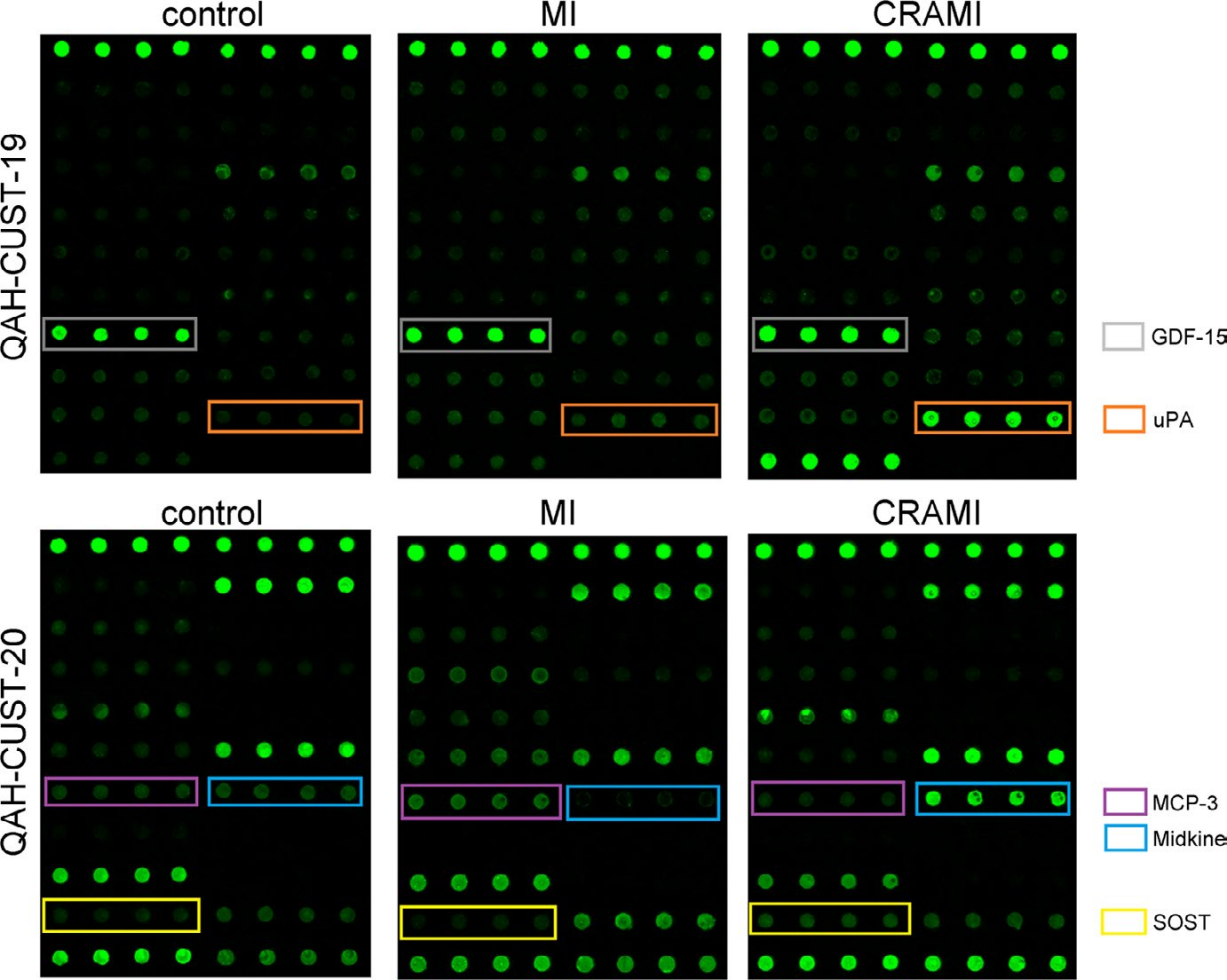
The antibody array profiles of the five specific proteins of cardiac remodelling after MI. The custom quantitative antibody array for validation was prepared in two arrays. The locations of the five proteins in the arrays are noted in coloured boxes, and the levels of these proteins are proportional to their fluorescence intensity. Corresponding antibodies of thirty‐nine proteins were printed in duplicate in these arrays. CRAMI group, cardiac remodelling after myocardial infarction; MI group, myocardial infarction without cardiac remodelling

To further validate these specific biomarkers, a quantitative antibody array was used and thirty‐nine proteins (including the eight specific proteins and thirty‐one other cytokines) only differential in the CRAMI vs MI group, or in CRAMI vs control group, and not in MI vs control group were detected again with more samples (Table 2). After the hoc Bonferroni analysis of one‐way ANOVA and Venn diagram analysis (Figure 4A), SOST, GDF‐15, uPA, MCP‐3 and MK were found to be simultaneously and significantly differentially expressed between CRAMI and MI group (*P* < .05), and between CRAMI and control group (*P* < .05), but not differential between MI and control group (*P* > .05). Further, they showed the same tendencies as those in the semi‐quantitative antibody array assay between the groups (Table 4 and Figure 4B). This suggests that these five cytokines may be robust and specific biomarkers of cardiac remodelling after MI. Additionally, their array profiles showed markedly different fluorescent intensities in the CRAMI group compared to that in the MI and control group, which were positively relevant to their expression levels, further confirming that these five proteins were uniquely differential in CRAMI (Figure 5).

**TABLE 4:** Specific biomarkers of cardiac remodelling after myocardial infarction from quantitative antibody array assay

	Control group	MI group	CRAMI group	MI vs Control		CRAMI vs Control		CRAMI vs MI	
	Mean ± SD	Mean ± SD	Mean ± SD	*P* value	FC	*P* value	FC	*P* value	FC
SOST	1.3 ± 3.1	19.7 ± 35.5	72.9 ± 80.7	1.000	15.277	.002	56.374	.004	3.690
uPA	100.0 ± 45.8	70.6 ± 56.8	209.7 ± 264.0	1.000	0.706	.009	2.097	.001	2.971
MK	340.1 ± 124.9	118.3 ± 223.0	2155.0 ± 752.7	.695	0.348	.000	6.337	.000	18.210
GDF‐15	367.4 ± 109.1	302.2 ± 95.8	520.6 ± 199.2	.682	0.823	.014	1.417	.000	1.723
MCP‐3	6.2 ± 3.5	8.2 ± 10.2	0.9 ± 0.6	.521	1.315	.000	0.145	.002	0.111

Abbreviations: CRAMI, cardiac remodelling after myocardial infarction; LVEDVi, left ventricular end‐diastolic volume index; LVEF, left ventricular ejection fraction; LVESVi, left ventricular end‐systolic volume index; MI, myocardial infarction without cardiac remodelling.

### 3.4 Correlation analysis between serum proteome and cardiac remodelling

**FIGURE 6:**
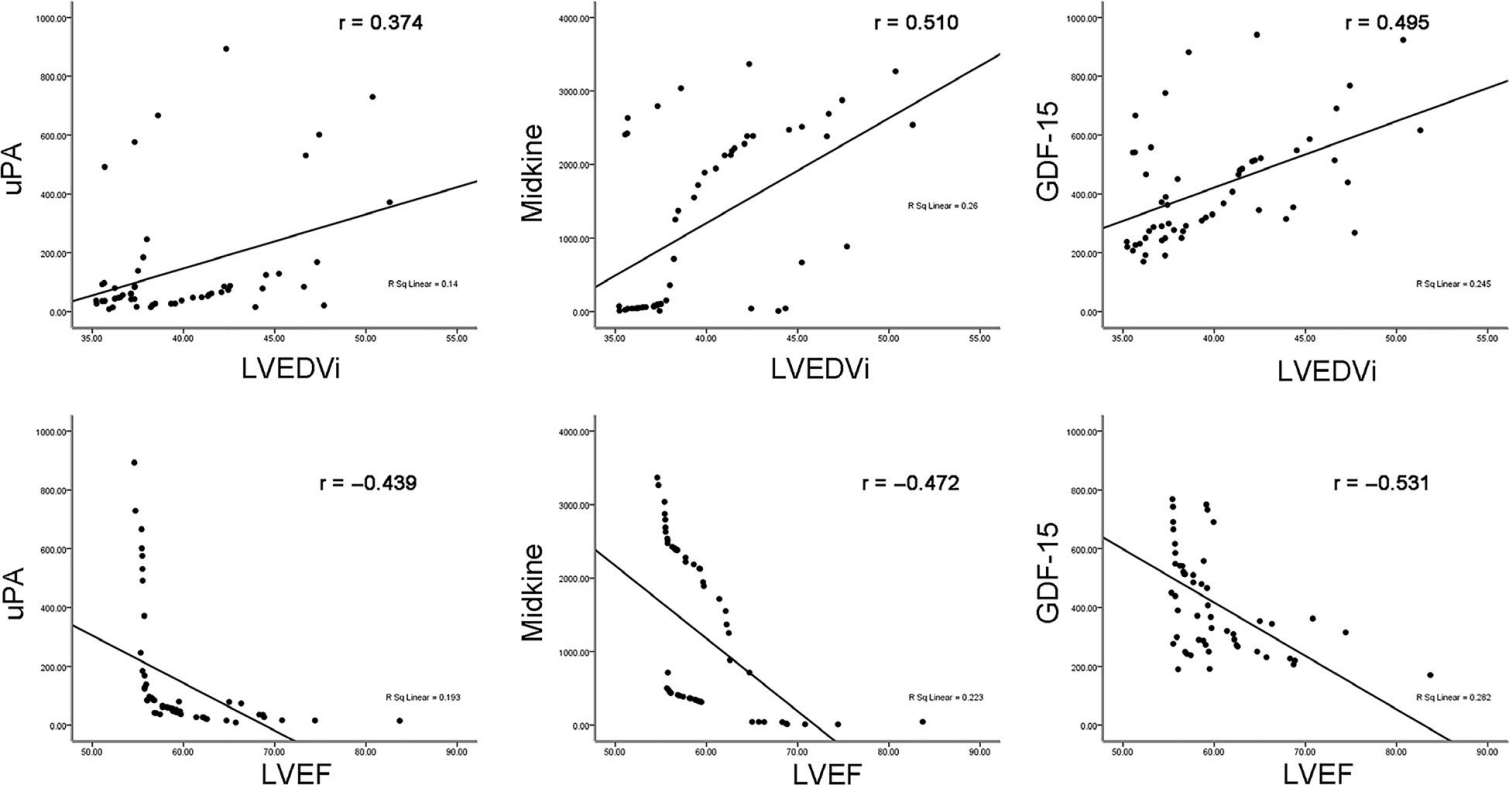
Correlation between serum proteome and cardiac remodelling echocardiographic parameters. Positive and negative correlations between the serum proteome and cardiac remodelling echocardiographic parameters, LVEDVi and LVEF

To further test the clinical utility of the five potential biomarkers identified in our study, we performed correlation analyses between serum proteomes and cardiac remodelling evaluated by LVEDVi and LVEF. The results demonstrated that LVEDVi positively correlated with the expression level of the three serum proteins (uPA, MK, GDF‐15). Notably, our results show that LVEF negatively correlated with these three serum proteins (uPA, MK, GDF‐15). The positive and negative correlation data are shown in Figure 6.

## 4 DISCUSSION

The identification of novel biomarkers for the prediction of cardiac remodelling is essential to develop timely reparative strategies against this adverse pathologic process.^21^ In the present exploratory case‐control study, to detect specific cytokines associated with the development of cardiac remodelling, serum samples were collected from fresh MI patients who were then followed up for 12 months for the monitoring of subsequent variations in ventricular structure and function. The results of a high‐throughput Human Cytokine Antibody Array that pre‐screened for 440 cytokines revealed eight cytokines (BMP‐2, SOST, CXCL14, GDF‐15, uPA, MCP‐3, MK and BMPR‐IB) that were found in MI patients with secondary cardiac remodelling one year later, using MI patients without cardiac remodelling development and healthy persons as control. Moreover, validation for these differential cytokines was performed using a custom quantitative antibody array, and the second screening identified five out of the eight cytokines (SOST, GDF‐15, uPA, MCP‐3, and MK) to be differentially expressed in the CRAMI group. Importantly, further statistical correlation analyses demonstrated that adverse cardiac remodelling was associated with three serum proteins (GDF‐15, uPA, and MK), suggesting that these three proteins may be specific serum biomarkers for the prediction of cardiac remodelling after MI at an early stage. Further bioinformatics analysis shows that these three proteins participate in the regulation of signalling receptor activity. Receptor activity is that when a molecule combines with an extracellular or intracellular messenger, a change in cell activity is initiated. This bioinformatics analysis result suggests that these three proteins may involve in the cardiac remodelling by regulating corresponding signalling receptor activity. Consistently, it has been shown that the progressive rearrangement or disruption of cell junctions enables the activation of ischaemic signalling leading towards heart failure.^22^

GDF‐15 is a transforming growth factor‐β cytokine superfamily member. GDF‐15 is produced by cardiac myocytes in response to mechanical stretch, ischaemia, oxidative stress and acute injury and plays a key role in the regulation of stress signals, inflammation and tissue repair after acute injury.^23^ GDF‐15 level is a strong predictor of long‐term mortality in patients with ST‐segment elevation myocardial infarction (STEMI).^24^ More recently, STEMI patients who subsequently developed LV remodelling at 12 months of follow‐up were found to have greater serum levels of GDF‐15 after the onset of STEMI symptoms, and therefore, GDF‐15 was identified as an independent predictor of LV remodelling.^25^ Consistently, the circulating levels of GDF‐15 were higher in MI patients who developed cardiac remodelling at 12 months of follow‐up, indicating that elevated GDF‐15 levels might promote the occurrence of cardiac remodelling and could be valuable as a robust and independent predictor of cardiac remodelling. Although bioinformatics analysis reveals the regulating signalling receptor activity function of GDF‐15, its exact function and the interaction with its receptor on the pathophysiological process of cardiac remodelling are still not clearly understood.

uPA is a plasmin activating pro‐matrix metalloproteinases in the cardiovascular system.^26^ uPA binding to its receptor (uPAR) plays a pivotal role in signalling functions which influence cell behaviours including cell adhesion, surface proteolysis, migration, proliferation, chemotaxis and cell extravasation.^27^ Elevation of uPA and uPAR is associated with the development of MI, dilated cardiomyopathy, cardiac fibrosis and heart failure.^28^ Furthermore, loss or inhibition of uPA is known to attenuate LV remodelling and dysfunction after acute pressure overload in mice, indicating that uPA could contribute to subsequent cardiac remodelling.^29^ Similarly, in this study, we found that elevated uPA levels were associated with the exacerbated post‐infarct pathological myocardial remodelling, indicating that uPA may serve as a predictor of cardiac remodelling in MI patients. In consistent, it was indicated that uPA affected the cardiac response to pressure overload is by migrating inflammatory cells and collagen‐producing cardiac fibroblasts to into the damaged myocardium.^30^ Therefore, it is likely that uPA aggravates adverse LV remodelling via binding to uPAR, subsequently causing progressive expansion of infarcted myocardium.

MK is a multifunctional heparin‐binding growth factor that mediates migration of inflammatory cell, neurite outgrowth, survival of embryonic neurons, and cell apoptosis via interactions with its receptors.^31^, ^32^ Although some studies have reported a relationship between MK and cardiac remodelling, the effect of MK on cardiac remodelling has been contradictory. Some studies reported that MK prevented cardiac remodelling after MI or congestive heart failure, while some studies found that MK deteriorated cardiac remodelling induced by pressure overload or chronic kidney disease using Midkine‐transgenic or knockout mice.^33^ Furthermore, Timotheus et al reported that MK was significantly increased in patients with heart failure compared to that in controls and was an independent predictor of cardiac events, whose incidence increased markedly with increase in MK levels.^34^ This is consistent with the present study which found that MI patients with elevated MK were likely to develop cardiac remodelling at 12 months of follow‐up, suggesting that MK is involved in the pathogenesis of cardiac remodelling. However, whether elevated MK levels in MI patient plays a protective role against cardiac remodelling, or an accelerated role in cardiac remodelling, and whether it is via interactions with its receptors, which needs further research.

Regrettably, there are no literatures about the potential relation of these three proteins in the pathological process, except that GDF‐15 is found to induce the invasiveness of gastric cancer cells by increasing uPA/uPAR system.^35^ This may suggest that up‐regulated GDF‐15 and MK might exacerbate cardiac remodelling by enhancing uPA/uPAR system responsible of inflammatory cells and cardiac fibroblasts for progressive expansion of infarcted myocardium.

Regarding the other identified proteins, recent studies have shown that SOST plays essential roles in bone formation, modelling and remodelling and is linked to bone physiology and cardiovascular disease.^36^ High levels of sclerostin are associated with calcification of the vascular tissue and cardiovascular mortality.^37^ Nevertheless, few studies have studied the relationship between SOST and cardiac remodelling after MI. Interestingly, the present study found high serum levels of SOST in MI patients who developed cardiac remodelling one year later, suggesting that SOST may play a pivotal role in development of vascular remodelling. MCP‐3 (monocyte chemotactic protein‐3) has potent chemotactic activities in recruitment of cells to inflammatory sites in a variety of inflammatory diseases.^38^ Soren Schenk et al found that MCP‐3 was transiently expressed in myocardial tissues after acute MI, which recruited mesenchymal stem cells to injured tissue sites and improved cardiac remodelling, suggesting that MCP‐3 plays a protective role against cardiac remodelling.^39^ Interestingly, we found that MI patients with lower circulating MCP‐3 levels were prone to subsequent cardiac remodelling one year later. These results suggested MCP‐3 might serve as a therapeutic drug against cardiac remodelling and low circulating levels of MCP‐3 may be a predictor of cardiac remodelling. Despite the fact that the present results could not find any relationship between SOST or MCP‐3 and cardiac remodelling echocardiographic parameters, further studies with a larger cohort may be necessary to establish the role of the two cytokines as original classifiers to discriminate cardiac remodelling in early phase of MI.

As many other novel efforts, our exploratory trial might also have certain limitations, due to the fact that the reported results have been generated only in a single‐centre and involved relatively small representative population. Therefore, a future more comprehensive work is needed to confirm our exploratory, but promising data, in a multi‐centre clinical trial with a larger number of participants. Also, the choice of proteins selected for microarrays assessment might not reflect all possible biomarkers that would be used as early predictors of cardiac remodelling after MI. Therefore, we cannot exclude the possibility that a number of other cytokines (not included in our panel) also participated in the process. In addition, we explored only a single time point for validation of the selected cytokine profile. Accordingly, yet there is a possibility that other profile of original biomarkers would also be detected at other time points in a further research.

## 5 CONCLUSION

In this study, an in‐depth proteomics approach was used to identify three specific biomarkers (GDF‐15, uPA and MK) as novel serum biomarkers for the early prediction of cardiac remodelling development after MI. More importantly, uPA and MK are reported for the first time for their critical roles in predicting adverse cardiac remodelling following MI. Regardless of the study limitations, including the small cohort size, the present results are of clinical significance and clearly support the validity of the approach. As the present work was a pilot study, the findings require further confirmation in a larger trial which would also appraise the value of these novel circulating biomarkers as therapeutic response in routine clinical practice.

## CONFLICT OF INTEREST

The authors declare that they have no competing interests.

## AUTHOR CONTRIBUTION

**Shuai MAO:** Data curation (lead); Formal analysis (lead); Funding acquisition (lead); Investigation (lead); Methodology (lead); Writing‐original draft (lead); Writing‐review & editing (lead). **Yubin Liang:** Methodology (supporting); Writing‐original draft (equal). **Peipei Chen:** Validation (equal). **Yuzhuo Zhang:** Methodology (equal). **Xin Yin:** Data curation (equal); Software (equal). **Minzhou Zhang:** Software (equal); Validation (equal); Visualization (equal); Writing‐review & editing (equal).

## Supporting information

Table S1

## Data Availability

All data generated or analysed during this study are included in this article.
